# Frequency of abnormal C-reactive protein concentrations in blood of dogs with hypoadrenocorticism

**DOI:** 10.1093/jvimsj/aalag054

**Published:** 2026-04-04

**Authors:** Miranda X Tong, Tiarni L Johnston, Carmel T Mooney, Anna Tebb, Spencer Tan, Corrin J Boyd, Giselle L Hosgood, Robert E Shiel

**Affiliations:** School of Veterinary Medicine, Murdoch University, Perth, WA 6150, Australia; Department of Internal Medicine, Western Australian Veterinary Emergency and Specialty, Perth, WA 6164, Australia; School of Veterinary Medicine, University College Dublin, Dublin 4, D04 W6F6, Ireland; Department of Internal Medicine, Western Australian Veterinary Emergency and Specialty, Perth, WA 6164, Australia; Advanced Vetcare Veterinary Centre, Bedok, 488692, Singapore; School of Veterinary Medicine, Murdoch University, Perth, WA 6150, Australia; School of Veterinary Medicine, Murdoch University, Perth, WA 6150, Australia; School of Veterinary Medicine, Murdoch University, Perth, WA 6150, Australia

**Keywords:** endocrinology, Addison’s disease, adrenal insufficiency, acute phase proteins

## Abstract

**Background:**

C-reactive protein (CRP) concentration in serum or plasma is high in humans during adrenal crisis due to multiple mechanisms, however, the frequency and clinical relevance of high CRP concentrations in dogs with hypoadrenocorticism are unknown.

**Hypothesis/Objectives:**

To determine the frequency of high CRP concentrations in serum or plasma of dogs with hypoadrenocorticism. We hypothesized that CRP concentrations are commonly above reference limit in dogs with hypoadrenocorticism with and without inflammatory comorbidities.

**Animals:**

Fifty-one dogs with hypoadrenocorticism presented to 2 referral centers between 2017 and 2023.

**Methods:**

Medical records were retrospectively reviewed for dogs diagnosed with and presenting for illness due to hypoadrenocorticism. Dogs were grouped according to identification of a concurrent inflammatory disease at the time of diagnosis, and whether electrolyte abnormalities were present. C-reactive protein concentrations were compared between groups.

**Results:**

Fifty-one dogs were included. C-reactive protein concentration in serum or plasma was above 10 mg/L in 38 of 51 (75%) cases. Median CRP concentration was 52.5 mg/L (range 8-191.4); this was not significantly different between dogs with (*n* = 14) or without (*n* = 37) a known inflammatory comorbidity (54.25 [8.0-191.4] and 44.0 [8-180.8] mg/L, respectively, *P* = .35), or between dogs with (*n* = 32) or without (*n* = 13) electrolyte abnormalities (48.25 [8.0-180.8] and 63.0 [8.0-191.4] mg/L, respectively, *P* = .51).

**Conclusions and clinical importance:**

High CRP concentrations in serum or plasma were frequently identified in dogs with hypoadrenocorticism, including dogs without recognized inflammatory comorbidities. Hypoadrenocorticism should be considered as a differential diagnosis in cases with high CRP concentrations and vague clinical signs. Extensive investigations for high CRP concentrations in dogs with hypoadrenocorticism might not be necessary without other supporting evidence of an inflammatory comorbidity.

## Introduction

C-reactive protein (CRP) is a positive acute phase protein synthesized primarily by hepatocytes and is induced by proinflammatory cytokines, predominantly interleukin (IL)-6.^1^^,^^2^ In dogs, high CRP concentrations are documented in a wide variety of infectious, inflammatory, and neoplastic conditions.^2-5^ It is generally regarded as a sensitive but non-specific marker of inflammation, increasing within hours of the inflammatory stimulus and returning to within reference interval within 1-2 weeks of resolution of inflammation.^6^ Measurement of CRP concentration is commonly used to aid diagnosis of inflammatory disease in dogs, and in some conditions can provide prognostic information.^2^^,^^7^

In human medicine, CRP is also a major acute phase protein useful for the diagnosis and monitoring of infectious and inflammatory conditions.^8^ Abnormal CRP concentrations can also be found in patients with adrenal insufficiency. CRP is identified as an independent marker of severe adrenal insufficiency (ie, adrenal crisis); higher CRP concentrations were documented during adrenal crisis compared to the chronic, stable, treated disease state.^9^ This was postulated to reflect, at least in part, a reduced glucocorticoid modulation of immune homeostasis resulting in higher proinflammatory cytokine expression, rather than solely the presence of inflammatory or infectious comorbidities. Another study identified higher CRP concentrations in patients diagnosed with adrenal insufficiency with fever compared to those that did not have fever; no infectious diseases were diagnosed in either group, however, the presence of non-infectious inflammatory comorbidities were not excluded.^10^ Those with fever had their diagnosis of adrenal insufficiency delayed on average 7.5 days, which the authors speculated was due to clinicians’ initial focus on identifying an infectious disease. Lack of glucocorticoid regulation of inflammatory cytokine expression was also proposed by these authors as the cause of the abnormal CRP concentrations and the resulting fever, suggesting that adrenal insufficiency itself might result in an inflammatory state. Experimental studies in mice demonstrate an enhanced expression of pro-inflammatory cytokines after adrenalectomy, possibly reflecting altered immunomodulatory response as a result of glucocorticoid deficiency.^11^ The role of glucocorticoids in modulating inflammatory cytokine expression, and the resulting cytokine release in adrenal insufficiency, is now widely recognized in human adrenal pathophysiology.^12^

There are contradictory reports regarding CRP concentrations in dogs with hypoadrenocorticism. In early reports, hypoadrenocorticism was listed as a disease that did not result in high CRP concentrations; however, these studies included dogs with a variety of infectious, inflammatory and neoplastic conditions, and the number of dogs with hypoadrenocorticism was low.^3^^,^^6^ A preliminary report has since described high CRP concentrations in 7 of 9 dogs with hypoadrenocorticism; however, the presence or absence of inflammatory comorbidities was not detailed.^13^ The expected values and significance of CRP concentrations in dogs with hypoadrenocorticism, and association with inflammatory comorbidities, require further evaluation.

There are multiple forms of hypoadrenocorticism and varying presentations that often mimic other diseases. The European Society of Veterinary Endocrinology has proposed common terminology via the Agreeing Language in Veterinary Endocrinology (ALIVE) project to describe the different forms based on pathophysiology and the presence of hyperkalemia, hyponatremia, or both.^14^ While the ACTH stimulation test has good sensitivity and specificity for the diagnosis of this disease, it relies on clinical suspicion to perform this test based on a combination of clinical and clinicopathological findings that are not pathognomonic. Given the range of clinical presentations and clinicopathological changes that might occur with this disease, recognition of an association with high CRP concentrations, if present, is important, as it might mean that search for other inflammatory/infectious disease is not always necessary in dogs with high CRP concentrations diagnosed with hypoadrenocorticism. The aims of this study were to determine the frequency of abnormal CRP concentrations in dogs with hypoadrenocorticism, and to explore the association between selected variables (including the presence or absence of comorbidities) and CRP concentration. It was hypothesized that CRP concentrations are commonly high in dogs with hypoadrenocorticism with and without inflammatory comorbidities.

## Materials and methods

Medical records from 2 referral centers (The Animal Hospital at Murdoch University [TAHMU], Murdoch, Western Australia and Western Australian Veterinary Emergency and Specialty [WAVES], Success, Western Australia) were retrospectively reviewed to identify dogs with hypoadrenocorticism between November, 2017 and August, 2023.

Dogs were included if they presented for an episode of illness due to hypoadrenocorticism, of any etiology, during the study period, and had CRP concentrations measured within 3 days of this presentation.

Dogs with naturally occurring hypoadrenocorticism were diagnosed by documenting inadequate cortisol production during an adrenocorticotropic hormone (ACTH) stimulation test (post-ACTH cortisol ≤ 55 nmol/L or lack of stimulation if > 55 nmol/L with other supportive evidence of hypoadrenocorticism),^15^ no prior administration of mitotane or trilostane, and no glucocorticoid and azole administration within the preceding 3 months of testing. Secondary hypoadrenocorticism was diagnosed based on results of ACTH stimulation testing as above, and endogenous ACTH concentrations < 10 pg/mL.^16^ Additionally, dogs with iatrogenic hypoadrenocorticism due to bilateral adrenalectomy or overtreatment with trilostane or mitotane were included if they had presented for an episode of illness due to hypoadrenocorticism. Illness due to hypoadrenocorticism was determined by a combination of supportive clinical signs such as lethargy, gastrointestinal signs, weakness, dehydration/hypovolemia or acute collapse,^16^ clinicopathological findings such as hyperkalemia or hyponatremia, and the assessment of the attending clinician as stated in the medical record. In cases of illness due to overtreatment of hypercortisolism, a post-ACTH cortisol concentration ≤ 40 nmol/L and clinical response to glucocorticoid supplementation were required.

Dogs that met the inclusion criteria were classified according to their diagnoses at presentation, adopting terminology proposed by the European Society of Veterinary Endocrinology via the ALIVE project. Diagnoses included (1) newly diagnosed primary hypoadrenocorticism with hyperkalemia, hyponatremia, or both (HHH), (2) newly diagnosed eukalemic and eunatremic hypoadrenocorticism (EEH), and those with electrolyte abnormalities not expected with mineralocorticoid deficiency (ie, hypokalemia or hypernatremia), (3) iatrogenic hypoadrenocorticism (bilateral adrenalectomy or overtreatment of hypercortisolism), and (4) recurrent hypoadrenal crisis (previously diagnosed hypoadrenocorticism presenting with signs of relapse). Each dog was only included once at the time of initial presentation during the study period.

Data recorded for each case included center, signalment, presentation type (emergency or routine), historical findings and their duration at presentation (signs present for < 21 days were classified as acute and recorded in days; signs present ≥ 21 days were classified as chronic but exact duration not specified), physical examination findings, prior medications, hematology, biochemistry and urinalysis results, blood gas analyses at presentation, initial CRP concentration, follow up CRP concentration if available, duration of hospitalization, outcome, and comorbidities. Timing of clinicopathological data (including CRP concentration) relative to presentation was recorded, as well as the laboratory where the testing was performed.

Concentrations of CRP were recorded as being performed in an in-house or external laboratory, with serum samples used for external testing and heparinized plasma for in-house testing. Method of measurement at the external laboratory, Vetpath Laboratory Services, was performed using the validated immunoturbidimetric species-specific Gentian Canine CRP Immunoassay (Gentian AS, Moss, Norway) on the Cobas pro 503 (Roche Diagnostics, Basel, Switzerland). In-house measurement was performed using the IDEXX Catalyst CRP Test sandwich immunoassay on the IDEXX Catalyst Dx (IDEXX Laboratories, Westbrook, USA). Reportable range of the Gentian assay was 8-300 mg/L, and 1-100 mg/L for the IDEXX assay. A diagnostic cut-off of 10 mg/L was used for both assays.^17^

The diagnosis of a comorbidity was determined by review of medical records (for newly diagnosed conditions) or history reported by the owner (for pre-existing conditions). The results of diagnostic tests used to support the diagnosis of a comorbidity were recorded. Comorbidities were characterized as inflammatory if the condition had been previously reported to be associated with high CRP concentrations.

### Statistical analyses

For descriptive statistics, all continuous variables were reported as median (range) to capture extremities of data, and categorical variables as frequency and percentages (%).

Cases were grouped based on the presence of a comorbidity and presence of electrolyte abnormalities at diagnosis. Comparison of CRP concentrations between groups were made using the Mann–Whitney U test. SPSS (IBM SPSS Statistics 29.0.0.0) was used for these analyses.

Multiple linear regression analysis was performed to explore any association between selected variables and CRP concentrations. Assessment of *R*^2^, adjusted *R*^2^, and Mallow’s p (C_p_) were used to select the simplest, best subset of variables to explain the variation in CRP concentration. Explanatory variables were evaluated for normality using visual assessment of histograms and Q-Q plots, and the Shapiro–Wilk test, with non-normal data log-transformed. Explanatory variables explored included neutrophil and lymphocyte counts, concentrations of glucose, cholesterol, albumin, sodium and potassium, CK activity, and the presence of comorbidities (yes/no) (Table S1). The simplest subset and the percentage explanation of variation in CRP concentration are reported. SAS v 9.4 (SAS Institute, Cary, NC) was used for this analysis.

## Results

A total of 51 cases met the inclusion criteria, 23 from TAHMU and 28 from WAVES. The median age at presentation was 5.7 (0.2-14.1) years. Median body weight was 17.3 (1.7-53.2) kg. There were 27 males (24 neutered) and 24 females (23 neutered). Thirty-one dogs were purebred of which the 6 most common breeds were Border collie (*n* = 5), standard poodle (*n* = 3), Alaskan malamute (*n* = 2), Chihuahua (*n* = 2), French bulldog (*n* = 2) and German Spitz (*n* = 2), and 20 were mixed breed, of which the most common was a poodle crossbreed (*n* = 20) (Table S2).

### Historical and physical examination data

Lethargy was the most frequently reported historical feature, followed by vomiting, hyporexia, diarrhea, and weakness/paresis. Of the 51 cases, 46 (90%) had acute development of clinical signs, a median of 5.3 days (1-14) before presentation. The most common abnormal physical examination findings reported at presentation were impaired mentation (44/51, 86%), dehydration (23/27, 85%), and abnormal pulse quality (31/51, 61%), however, not all variables were reported in each case (Table S3).

### Clinicopathologic data

Hematology and serum biochemistry results, including CRP concentrations, are presented in Table S4. The first measurement of CRP concentrations was performed on the day of presentation in 34 (67%) cases, on the day after presentation in 14 (28%) cases, 2 days after presentation in 4 (8%) cases, and 3 days after presentation in 1 (2%) case. Three cases had CRP concentrations measured in-house only and all were within reportable range of the IDEXX assay.

**Figure 1:**
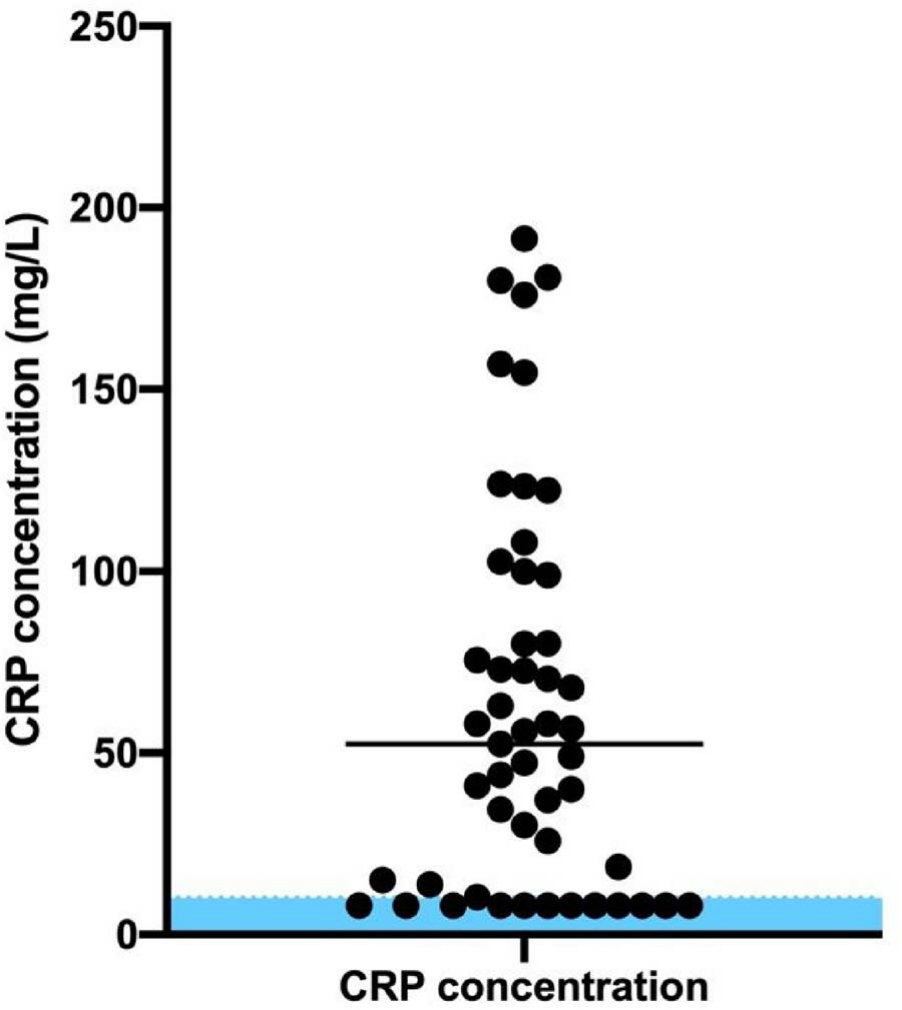
Distribution of CRP concentrations in 51 dogs with hypoadrenocorticism. Shaded area represents reference interval CRP concentrations (<10 mg/L). The horizontal line denotes the median CRP concentration.

The median CRP concentration of all cases was 52.5 mg/L (8-191.4) (Figure 1). In 38 (75%) cases, CRP concentration was above the diagnostic cut-off of 10 mg/L. Cases with normal CRP concentrations all measured below the lower limit of detection of the Gentian assay and were assigned a value of 8 mg/L. When only including cases that had CRP concentrations measured on the day of presentation (before any newly prescribed therapy), the proportion of high CRP concentrations was 65% (22/34), compared to 82% (14/17) of cases that first had CRP concentrations measured 1-3 days after presentation—this included 12 of 14 (86%), 1 of 1, and 2 of 2 dogs with high CRP concentrations on days 1, 2, and 3 after presentation, respectively.

Five dogs had a follow-up CRP concentration measured at a subsequent visit after discharge, with a median of 5 days (3-10) between CRP measurements. Four of the five dogs had high CRP concentrations at presentation, and in all 4 dogs, CRP concentrations were lower at the subsequent visit. The median change in CRP concentrations was 32 mg/L (24.5-125.4). The fifth dog had undetectable CRP concentrations at both presentation and the subsequent visit.

### Comorbidities

Thirty-five (69%) dogs had no comorbidities recorded and 16 (31%) had a comorbidity recorded (Table 1).

**Table 1:** Recorded comorbidities in dogs with hypoadrenocorticism. “Inflammatory comorbidity” indicates disease known to be associated with high CRP concentrations.^2^^,^^3^^,^^6^

**Comorbidity**	**Number**
**Inflammatory comorbidity**	
**Aspiration pneumonia**	2
** Urinary tract infection/subclinical bacteriuria**	2
** Inflammatory primary hepatopathy**	1
** Chronic enteropathy**	2
** Acute pancreatitis**	1
** Pyoderma**	2
** Dental disease**	3
** Immune-mediated thrombocytopenia**	1
** Abnormal DGGR-lipase activity**	5
**Non-inflammatory comorbidity**	
** Diabetes mellitus**	1
** Thrombocytopenia—unknown cause**	1
** Hypothyroidism**	1

**Figure 2:**
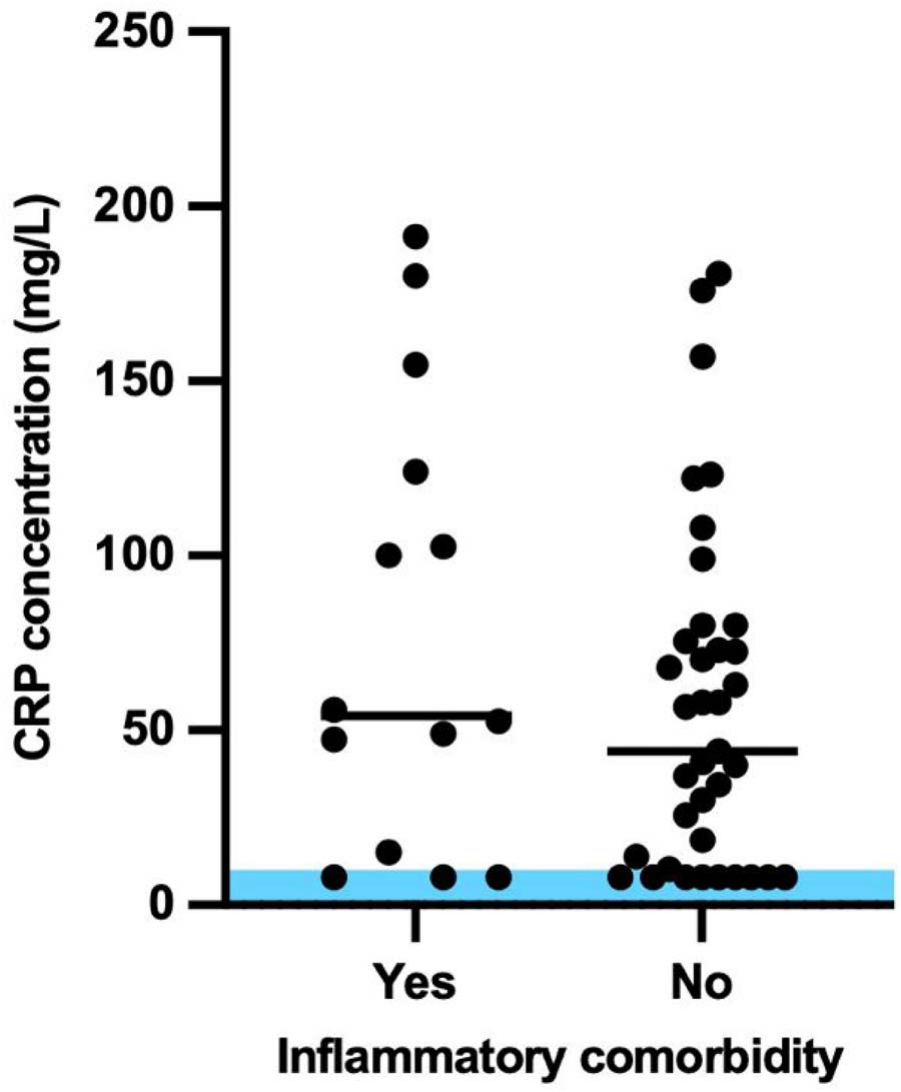
Distribution of CRP concentrations in dogs with hypoadrenocorticism with and without a documented inflammatory comorbidity. Shaded area represents reference interval CRP concentrations (<10 mg/L). The horizontal lines denote the median CRP concentration.

Median CRP concentration in dogs with known inflammatory comorbidities (*n* = 14) was 54.25 mg/L (8.0-191.4 mg/L), and in dogs with no known comorbidity or non-inflammatory comorbidity only (*n* = 37) was 44.0 mg/L (8.0-180.8 mg/L); there was no statistically significant difference between the 2 groups (*P* = 0.35) (Figure 2).

A total of 12 different comorbidities were recorded of which 3 were considered non-inflammatory. Eight dogs had more than one comorbidity. Both dogs diagnosed with aspiration pneumonia had thoracic radiographs performed; however, in 1 dog there was no radiographic evidence of an alveolar pattern. Early aspiration pneumonia was still suspected in this dog based on the presence of abnormal respiratory effort, pyrexia of 40.4 C, number of b-lines on point-of-care thoracic ultrasound, and response to antimicrobial therapy. A positive urine culture was identified in 2 of 16 dogs in which it was performed, 1 with non-hemolytic *Escherichia coli* and 1 with *Klebsiella pneumoniae*. The former had reported inappropriate urination before presentation, while urination pattern was not described in the latter case. Acute pancreatitis was diagnosed in 1 dog based on ultrasonographic features of mild peripancreatic hyperechogenicity, although DGGR-lipase activity was not high (15 U/L). A further 5 of 46 dogs (11%) had high DGGR-lipase activity, although pancreatitis was not suspected clinically by the attending clinician. Immune-mediated thrombocytopenia was diagnosed concurrently in 1 dog at the time of presentation, based on persistent severe thrombocytopenia (manual platelet count 10 × 10^9^/L) and development of petechiae. Immunosuppressive corticosteroid therapy was commenced but the dog was euthanized before a response to treatment could be seen. One other dog also had a severe thrombocytopenia of unknown cause (manual platelet count 10 × 10^9^/L) with a concurrent coagulopathy evident on thromboelastometry. A consumptive process was suspected but this was not confirmed despite further investigations (abdominal ultrasound, thoracic and abdominal computed tomography, vector-borne disease testing). Anticoagulant therapy was commenced, and platelet numbers and coagulation status improved within 1 week.

### Form of hypoadrenocorticism and outcome

All dogs with naturally occurring primary hypoadrenocorticism had post-ACTH stimulation cortisol concentration≤  55 nmol/L. Thirty-two dogs (63%) were newly diagnosed with HHH at presentation. Thirteen dogs (26%) were newly diagnosed with EEH at presentation. Of these, one dog had suspected secondary hypoadrenocorticism based on a baseline cortisol concentration of 49.9 nmol/L with an ACTH-stimulated cortisol concentration of 69.4 nmol/L, and an endogenous ACTH concentration less than the limit of detection of the assay (5 pg/mL). Aldosterone concentrations in this dog pre- and post-ACTH stimulation were 653 and 713 pmol/L, respectively. This dog also had the thrombocytopenia of unknown cause as described above, which resolved without need for immunosuppressive therapy. Adequate aldosterone concentrations were also documented in 2 dogs with EEH.

Three dogs (6%) were classified as iatrogenic hypoadrenocorticism—1 dog had bilateral adrenalectomy 3 years before presentation and was receiving prednisolone and desoxycorticosterone pivalate. This dog had high CRP concentrations of 75.60 mg/L with no documented comorbidity. Two dogs were receiving trilostane therapy for hyperadrenocorticism, and ACTH stimulation testing confirmed overtreatment at the time of presentation (pre- and post-ACTH cortisol concentrations both ≤ 40 nmol/L). One of these cases had high CRP concentrations (49 mg/L) at presentation and high DGGR-lipase activity, while the other had normal CRP concentrations despite a concurrent pyoderma.

Three dogs (6%) with previously diagnosed hypoadrenocorticism presented due to recurrence of clinical signs. The initial presentation of these dogs was before the start date of the current study. These dogs had clinical signs compatible with hypoadrenocorticism, sodium-to-potassium ratios < 27:1, and responded rapidly to therapy including additional glucocorticoid/mineralocorticoid supplementation. Two of these dogs had normal CRP concentrations at presentation, while the third had a markedly high CRP concentration (80 mg/L) with no documented inflammatory comorbidity.

**Figure 3:**
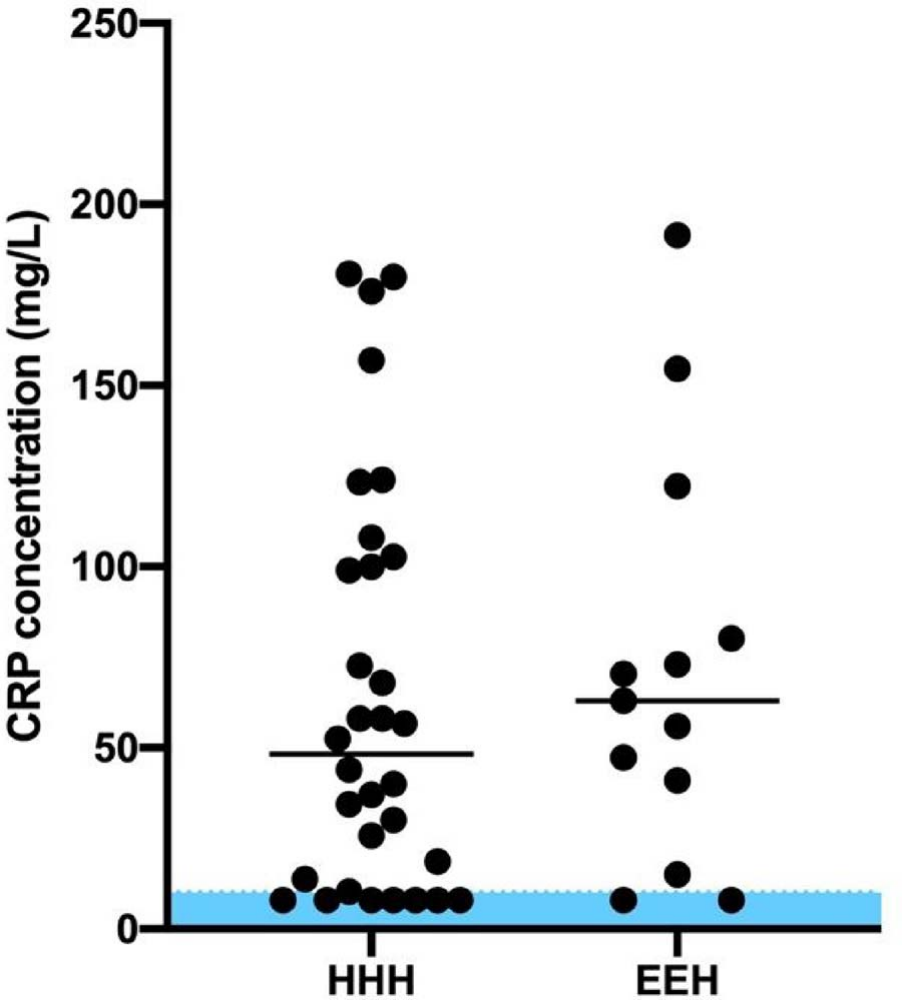
Distribution of CRP concentrations in dogs with newly diagnosed primary hypoadrenocorticism with hyperkalemia, hyponatremia, or both (HHH) and newly diagnosed eukalemic and eunatremic hypoadrenocorticism (EEH). Shaded area represents reference interval CRP concentrations (<10 mg/L). The horizontal lines denote the median CRP concentration.

Of the dogs with newly diagnosed hypoadrenocorticism, median CRP concentrations were not statistically different (*P* = .51) between dogs with HHH (48.25 mg/L [8.0-180.8]) and dogs with EEH (63.0 mg/L [8.0-191.4]) (Figure 3).

Six dogs (12%) were euthanized during hospitalization or within 7 days after discharge; no dogs died spontaneously. Three of the dogs that were euthanized had a concurrent comorbidity and 3 were diagnosed with hypoadrenocorticism alone. Five of the 6 dogs had high CRP concentrations, ranging from 70 to 124 mg/L. Of the dogs that survived to discharge, the median duration of hospitalization was 2 days (0-10).

### Regression analysis

All selected continuous variables were normally distributed, either with or without log transformation. The best single variable subset that explained the variation in CRP was CK concentration, explaining 15% of the variation in CRP (*R*^2^ = 0.1516). This explanation of variation in CRP was increased to 21% with the addition of blood glucose concentration (2 variable model, Adjusted *R*^2^ = 0.2097) and subsequently to 24% with the addition of neutrophil concentration (3 variable model, Adjusted *R*^2^ = 0.2418) or to 25% with the addition of cholesterol concentration (3 variable model, Adjusted *R*^2^ = 0.2468). No explanation in variance was gained with 4 variable models.

## Discussion

In this study, 75% of dogs with hypoadrenocorticism had abnormally high CRP concentrations in serum or plasma. In many cases, the magnitude of the abnormality was marked, with a median CRP concentration of 52.5 mg/L (8-191.4). This is similar to the frequency and magnitude of abnormal CRP concentrations previously reported in a single abstract.^13^ In that study, CRP concentrations were high in 7 of 9 (78%) dogs diagnosed with hypoadrenocorticism, with a median CRP concentration of 62.2 mg/L (interquartile range: 20.45-89.66 mg/L). However, unlike the previous report, the current study documented that CRP concentrations were high in dogs with and without apparent inflammatory comorbidities, and in dogs with both EEH and HHH.

Dogs with hypoadrenocorticism might have a greater risk of developing secondary inflammatory complications. Vomiting or regurgitation might lead to aspiration pneumonia. Gastrointestinal bacterial translocation^18^^,^^19^ or secondary systemic inflammatory response syndrome/sepsis might develop in animals with severe intestinal involvement.^5^ Hypovolemia could also result in development of pancreatitis or tissue hypoxia leading to a systemic inflammatory state.^20^ Conversely, it is recognized that acute clinical signs of hypoadrenocorticism might be precipitated by a stressful event,^21^ and it is possible that acute inflammation or infection might have been a stressor in some dogs. Although not specifically recognized in dogs, infection is a major risk factor for the development of adrenal crisis in humans.^22^^,^^23^ The presence of coincidental infectious or inflammatory disease cannot be excluded in all cases. Concurrent sources of inflammation other than hypoadrenocorticism is a less likely cause of high CRP concentrations because comorbid inflammatory diseases were only identified in approximately one third of cases, and there was no association between the presence of inflammatory comorbidities and CRP concentration on regression analysis. It is possible that not all inflammatory comorbidities were identified, particularly if associated clinical signs were mild or masked by the presence of hypoadrenocorticism. The retrospective nature of the study limited definitive exclusion of comorbidities in each case; additional diagnostic investigations beyond the inclusion criteria were performed at the discretion of the attending clinician. However, in 32 of 35 dogs with no inflammatory comorbidity recorded, clinical signs resolved by discharge while only receiving supportive therapy (ie, intravenous fluid therapy, anti-nausea medications, pain relief) in addition to glucocorticoid and (if indicated) mineralocorticoid supplementation, suggesting that any undetected inflammatory comorbidity had limited clinical relevance.

Although biopsy is rarely performed, the most common cause of canine primary hypoadrenocorticism is lymphoplasmacytic adrenalitis.^24^ While CRP concentrations are abnormal in a number of autoimmune diseases,^2^ most affected dogs have severe adrenocortical atrophy by the time of diagnosis.^24^^,^^25^ Due to this, and the small volume of affected tissue, the authors believe it is unlikely that adrenalitis alone is responsible for the high CRP concentrations. Furthermore, the dog with bilateral adrenalectomy in this study had high CRP concentrations that could not be attributed to another inflammatory cause.

A mechanism for high CRP concentrations directly related to glucocorticoid deficiency was proposed by Katabami et al. when CRP concentrations were surprisingly found to be an independent predictor of adrenal crisis in human patients.^9^ Proinflammatory cytokines involved in the induction of CRP, such as IL-1, IL-6, and tumor necrosis factor-α, have been shown to activate the hypothalamic–pituitary–adrenal axis,^26^^,^^27^ increasing the production of cortisol. This is part of a complex physiological neuroendocrine network that modulates the immune system to ensure an appropriate but not excessive response.^28^ Glucocorticoids modulate the immune system by promoting T-helper(Th)2 type cytokines, and a deficiency of cortisol due to inadequate production might result in greater sensitivity and production of Th-2 type inflammatory cytokines, leading to a higher production of CRP.^9^^,^^29^^,^^30^ High IL-6, and to a lesser extent TNF-α and IL-1 concentrations are also described in humans with primary and secondary hypocortisolism, suggesting that hypocortisolism might contribute to the development of a pro-inflammatory state.^31^^,^^32^ In the current study, CRP concentrations were high in a similar proportion of dogs with EEH and HHH. This supports that glucocorticoid deficiency (and not mineralocorticoid deficiency) is responsible for the high CRP concentrations.

No strong independent predictors of CRP concentration were identified in the current study. A 3-variable model including CK activity and glucose concentration as well as either neutrophil count or cholesterol concentration explained, at most, only 25% of the variation in CRP concentration. Glucose and cholesterol concentrations, as well as neutrophil counts, are known to be associated with glucocorticoid deficiency, although not abnormal in all cases. Mature neutrophilia is reported in approximately one third of dogs with hypoadrenocorticism, and band neutrophilia in approximately 15% of cases, in the absence of an identified infectious/inflammatory comorbidity.^15^^,^^16^ Similar to the high CRP concentrations, this could reflect the presence of an unrecognized inflammatory comorbidity, or a reduced immunomodulatory effect of corticosteroids on neutrophil function.^33^ The effect of CK activity might be explained by prolonged recumbency, and thus more severe presentation, in these cases. In human medicine, CRP concentrations are more likely to be high in patients with adrenal crisis compared to those presenting with more chronic disease. The absence of defined criteria to identify adrenal crisis in veterinary medicine, and the retrospective nature of the current study, made this difficult to assess, and therefore acuity of presentation was not included in the regression analysis. Overall, only a weak association between the explanatory variables included in the models and CRP concentration was identified.

The high frequency of high CRP concentrations in dogs with hypoadrenocorticism without apparent inflammatory comorbidities is important to recognize during the clinical evaluation of cases. Dogs with hypoadrenocorticism often have vague clinical signs, and clinicopathological changes might also be non-specific, especially in dogs with glucocorticoid-only deficiency. Therefore, failure to consider hypoadrenocorticism as a differential diagnosis could result in extensive investigation to determine the underlying cause. Likewise, documenting a high CRP concentration alone as evidence of inflammation should not prompt further investigations in a dog with hypoadrenocorticism, especially if there is a rapid and complete response to therapy. However, further testing might still be indicated in the presence of additional clinical signs of inflammation (such as pyrexia, which has not been found to be associated with hypoadrenocorticism in dogs), or evidence of involvement of other systems (such respiratory distress). The value of CRP measurement in the diagnosis of hypoadrenocorticism could not be assessed in the current study in the absence of a control group.

Limitations of this retrospective study include the inability to standardize the timing and method of clinicopathological testing. Not all dogs had CRP measurement on the day of initial presentation, with sample collection delayed up to 3 days in some cases. However, this limitation reflects the timing of CRP measurement in most clinical practices, with more extensive clinicopathological testing often performed after initial stabilization. Treatments administered before CRP measurement, including corticosteroids, could have decreased CRP concentrations.^34^ The exact timing of corticosteroid commencement in relation to sample collection for CRP concentrations was not always clear from medical record review, and this, along with patients presenting in recurrent hypoadrenal crisis already receiving corticosteroids, limited ability to compare CRP concentrations between dogs that were receiving corticosteroids at time of CRP measurement and those that were not. However, the relatively long half-life of CRP in dogs of 19 hours^2^ make it unlikely that moderately or markedly high CRP values would rapidly normalize within this timeframe, even if the inflammatory disease immediately resolved with corticosteroid therapy. This is supported by the observation that the proportion of cases with high CRP concentration was similar in dogs with immediate and delayed measurement, despite prior administration of exogenous corticosteroids to most cases in the latter group. Due to the limited numbers in the groups within CRP measurement on days 2 and 3, statistical comparisons were not made between the groups. Similarly, in human medicine, CRP is an independent predictor of adrenal crisis, regardless of prior corticosteroid therapy.^9^

Most clinicopathological testing was performed at a single external laboratory (used by both referral centers at the time of the study); however, samples analyzed using in-house instruments were also included. While the IDEXX Catalyst CRP assay does have good agreement with the Gentian CRP assay, results might not be directly interchangeable.^17^ Results from both assays were included to reflect the frequent measurement of this parameter using point-of-care instruments, especially when cases are presented outside of standard laboratory operating hours. In addition, CRP concentrations were measured solely on the IDEXX Catalyst assay in only 3 cases, which would not be expected to have significant impact on the results.

In conclusion, CRP concentrations are frequently high in dogs with hypoadrenocorticism without apparent inflammatory comorbidities. In dogs with vague clinical signs, high CRP concentrations—once other causes of inflammation have been excluded—might lead the clinician to include hypoadrenocorticism among the possible causes. In such cases, it is advisable to initiate a diagnostic work-up aimed at identifying the underlying endocrine disorder.

## Supplementary Material

CRP_hypoadrenocorticism_supplementary_data_1_aalag054

CRP_hypoadrenocorticism_Supplementary_data_2_aalag054

CRP_hypoadrenocorticism_Supplementary_data_3_aalag054

CRP_hypoadrenocorticism_supplementary_data_4_aalag054

## Off-label antimicrobial declaration

Authors declare no off-label use of antimicrobials.

## Institutional animal care and use committee or other approval declaration

Authors declare no institutional animal care and use committee or other approval was needed.

## Abbreviations

AST: aspartate aminotransferase
ALT: alanine transaminase
BUN: blood urea nitrogen
CRP: C-reactive protein
CK: creatine kinase
DGGR-lipase: 1,2-o-dilauryl-rac-glycero-3-glutaric acid-(6′-methylresorufin) ester-lipase
IL: interleukin
EEH: eukalemic and eunatremic hypoadrenocorticism
HHH: hypoadrenocorticism with hyperkalemia hyponatremia or both
Na: K: sodium-to-potassium
PvCO_2_: venous partial pressure of carbon dioxide
TAHMU: The Animal Hospital at Murdoch University
USG: urine specific gravity
WAVES: Western Australian Veterinary Emergency and Specialty
