# Supplementary material for: Frequency of abnormal C-reactive protein concentrations in blood of dogs with hypoadrenocorticism
Source: J Vet Intern Med. 2026 Apr 4;40(2):aalag054. doi: 10.1093/jvimsj/aalag054 (PMC13050043; doi:10.1093/jvimsj/aalag054)
Supplement: CRP_hypoadrenocorticism_Supplementary_data_2_aalag054 [file crp_hypoadrenocorticism_supplementary_data_2_aalag054.docx]

Supplementary table 2. Breed distribution of 51 dogs with hypoadrenocorticism.

| **Breed** | **Number (%)** |
| --- | --- |
| Mixed breed  Poodle cross  Staffordshire Bull Terrier cross  Maltese cross  Jack Russell Terrier cross  Chihuahua cross  Rottweiler cross  Beagle cross  Labrador cross  German Shorthaired Pointer cross  Cavalier King Charles Spaniel cross | 20 (39)  5 (10)  4 (8)  3 (6)  2 (4)  1 (2)  1 (2)  1 (2)  1 (2)  1 (2)  1 (2) |
| Border Collie | 5 (10) |
| Standard Poodle | 3 (6) |
| Alaskan Malamute | 2 (4) |
| Chihuahua | 2 (4) |
| French Bulldog | 2 (4) |
| German Spitz | 2 (4) |
| Airedale Terrier | 1 (2) |
| Beagle | 1 (2) |
| Corgi | 1 (2) |
| Dachshund | 1 (2) |
| Dalmatian | 1 (2) |
| Dogue de Bordeaux | 1 (2) |
| German Shepherd Dog | 1 (2) |
| Great Dane | 1 (2) |
| Brussels Griffon | 1 (2) |
| Jack Russell Terrier | 1 (2) |
| Labrador | 1 (2) |
| Mastiff | 1 (2) |
| Rottweiler | 1 (2) |
| Staffordshire Bull Terrier | 1 (2) |
| Chinese Crested Dog | 1 (2) |
