# Supplementary material for: Frequency of abnormal C-reactive protein concentrations in blood of dogs with hypoadrenocorticism
Source: J Vet Intern Med. 2026 Apr 4;40(2):aalag054. doi: 10.1093/jvimsj/aalag054 (PMC13050043; doi:10.1093/jvimsj/aalag054)
Supplement: CRP_hypoadrenocorticism_Supplementary_data_3_aalag054 [file crp_hypoadrenocorticism_supplementary_data_3_aalag054.docx]

| **Physical examination parameter** | **Number (%)** | **Total number recorded** |
| --- | --- | --- |
| Demeanour  Bright, alert and responsive  Quiet, alert and response  Dull  Obtunded  Stuporous  Comatose | 6 (12)  31 (62)  6 (12)  5 (10)  2 (4)  0 (0) | 50 |
| Respiratory effort  Normal  Mild increase  Moderate increase  Severe increase | 46 (90)  2 (4)  3 (6)  0 (0.0) | 51 |
| Pulse quality  Hypokinetic  Normokinetic  Hyperkinetic | 30 (59)  20 (39)  1 (2) | 51 |
| Mucous membrane colour  Pale  Pale pink  Pink  Injected  Cyanotic | 5 (10)  3 (6)  33 (65)  10 (20)  0 (0) | 51 |
| Hydration status  Euhydrated  Mild dehydration (<5%)  Moderate (~8%)  Severe (>10%) | 4 (15)  12 (44)  10 (37)  1 (4) | 27 |
| Capillary refill time  < 1 second  1 second  1.5 seconds  2 seconds  > 2 seconds | 0 (0)  11 (22)  6 (12)  23 (46)  10 (20) | 50 |
| Temperature  Hypothermic (<37.5 °C)  Normothermic (37.5 °C – 39.2 °C)  Hyperthermic (> 39.2 °C) | 19 (39)  25 (51)  5 (10) | 49 |

Supplementary table 3. Summary of physical examination parameters recorded in 51 dogs presenting with illness due to hypoadrenocorticism.
