# Supplementary material for: Frequency of abnormal C-reactive protein concentrations in blood of dogs with hypoadrenocorticism
Source: J Vet Intern Med. 2026 Apr 4;40(2):aalag054. doi: 10.1093/jvimsj/aalag054 (PMC13050043; doi:10.1093/jvimsj/aalag054)
Supplement: CRP_hypoadrenocorticism_supplementary_data_4_aalag054 [file crp_hypoadrenocorticism_supplementary_data_4_aalag054.docx]

| **Parameter** | **Median (range)** | **Number (%) below RI** | **Number (%) within RI** | **Number (%) above RI** | **Total number** | **Reference interval (RI)** |
| --- | --- | --- | --- | --- | --- | --- |
| Hematocrit (L/L) | 0.485 (0.24-0.71) | 12 (24) | 23 (46) | 15 (30) | 50 | 0.37-0.66 |
| Hemoglobin (g/L) | 170 (77-260) | 9 (18) | 29 (57) | 13 (25) | 51 | 120-180 |
| Red blood cell count (x10^12/L) | 7.4 (3.4-10.9) | 8 (16) | 30 (59) | 13 (25) | 51 | 5.5-8.5 |
| Reticulocyte count (x10^9/L) | 31 (0-350) | n/a | 46 (94) | 3 (6) | 49 | 0-95 |
| Total white blood cell count (x10^9/L) | 12.3 (4.4-61.8) | 2 (4) | 37 (72) | 12 (24 | 51 | 6.0-17.0 |
| Neutrophil count (x10^9/L) | 8.54 (3.45-49.56) | 0 (0) | 37 (74) | 13 (26) | 50 | 3.00-11.50 |
| Band neutrophil count (x10^9/L) | 0.00 (0.00-10.00) | n/a | 45 (90) | 5 (10) | 50 | 0.00-0.30 |
| Lymphocyte count (x10^9/L) | 3.06 (0.39-9.90) | 3 (6) | 44 (87) | 4 (8) | 51 | 1.00-4.80 |
| Eosinophil count (x10^9/L) | 0.50 (0.00-3.28) | 9 (18) | 36 (70) | 6 (12) | 51 | 0.10-1.25 |
| Platelet count (automated) (x10^9/L) | 238 (0-538) | 17 (33.3) | 34 (66.7) | 0 (0) | 51 | 200-900 |
| Creatine kinase (U/L) | 546 (86-59171) | 0 (0) | 13 (26.5) | 36 (73.5) | 49 | 47-228 |
| Aspartate aminotransferase (U/L) | 66 (23-3482) | 0 (0) | 19 (40) | 28 (30) | 47 | 10-60 |
| Alanine transaminase (U/L) | 58 (19-365) | 1 (2) | 47 (92) | 3 (6) | 51 | 21-142 |
| Alkaline phosphatase (U/L) | 37 (8-436) | 7 (14) | 41 (80) | 3 (6) | 51 | 20-184 |
| Gamma-glutamyl transferase (U/L) | 1 (0-10) | 4 (8) | 45 (90) | 1 (2) | 49 | 1-8 |
| Total bilirubin (umol/L) | 2 (0-34) | 0 (0) | 49 (98) | 1 (2) | 50 | 2-17 |
| Amylase (U/L) | 1079 (112-2849) | 1 (2) | 36 (71) | 14 (27) | 51 | 340-1400 |
| DGGR-lipase (U/L) | 21.5 (7-671) | n/a | 41 (89) | 5 (11) | 46 | 0-90 |
| Cholesterol (mmol/L) | 3.5 (1.2-11.2) | 23 (47) | 22 (45) | 4 (8) | 49 | 3.3-6.9 |
| Urea (mmol/L) | 11.3 (3.2-61.4) | 3 (6) | 19 (37) | 29 (57) | 51 | 3.6-10.0 |
| Creatinine (umol/L) | 141.5 (34-686) | 1 (2) | 21 (42) | 28 (56) | 50 | 44-132 |
| Sodium (mmol/L) | 139 (119-157) | 25 (49) | 25 (49) | 1 (2) | 51 | 139-154 |
| Potassium (mmol/L) | 5 (3.4-10.0) | 0 (0) | 16 (31) | 35 (69) | 51 | 3.4-5.3 |
| Na:K ratio | 21.75 (10.12-42.94) | n/a | 34 (66.6) | 17 (33.3) | 51 | <27 |
| Chloride (mmol/L) | 105 (87-132) | 14 (27) | 35 (69) | 2 (4) | 51 | 99-120 |
| Glucose (mmol/L) | 4.21. (1.6-19.1) | 9 (18) | 36 (74) | 4 (8) | 49 | 3.6-6.8 |
| Albumin (g/L) | 29 (12-40) | 17 (34) | 33 (65) | 1 (2) | 51 | 24-38 |
| Globulin (g/L) | 33 (17-56) | 15 (30) | 32 (63) | 4 (8) | 51 | 28-44 |
| Total calcium (mmol/L) | 2.60 (1.82-4.33) | 11 (22) | 26 (40) | 14 (27) | 51 | 2.20.2.80 |
| Phosphorus (mmol/L) | 1.92 (0.70-5.97) | 3 (6) | 28 (55) | 20 (39) | 51 | 0.80-2.20 |
| pH | 7.305 (7.150-7.410) | 27 (59) | 19 (41) | 0 (0) | 46 | 7.320-7.430 |
| PvCO_2_ (mmHg) | 36.25 (20.0-51.4) | 33 (72) | 9 (20) | 4 (9) | 46 | 40.0-46.0 |
| Bicarbonate (mmol/L) | 17.5 (9.8-38.8) | 31 (82) | 5 (13) | 2 (5) | 38 | 22.0-24.0 |
| Lactate (mmol/L) | 1.4 (0.5-4.7) | n/a | 36 (82) | 8 (18) | 44 | <2.0 |
| Ionised calcium (mmol/L) | 1.33 (1.10-1.94) | 9 (20) | 29 (66) | 6 (14) | 44 | 1.25-1.50 |
| C-reactive protein (mg/L) | 52.5 (8-191.4) | n/a | 13 (25) | 38 (75) | 51 | <10 |

Supplementary table 4. Summary of hematology, serum biochemistry and venous blood gas analyses in 51 dogs presenting with illness due to hypoadrenocorticism.
